# Tumor Primary Location May Affect Metastasis Pattern for Patients with Stage IV NSCLC: A Population-Based Study

**DOI:** 10.1155/2020/4784701

**Published:** 2020-07-09

**Authors:** Qinge Shan, Zhenxiang Li, Jiamao Lin, Jun Guo, Xiao Han, Xinyu Song, Haiyong Wang, Zhehai Wang

**Affiliations:** ^1^Department of Internal Medicine-Oncology, Shandong Cancer Hospital and Institute, Shandong First Medical University and Shandong Academy of Medical Sciences, Jinan, Shandong 250117, China; ^2^Department of Radiation Oncology, Shandong Cancer Hospital and Institute, Shandong First Medical University and Shandong Academy of Medical Sciences, Jinan, Shandong 250117, China

## Abstract

**Background:**

Most patients with nonsmall cell lung cancer (NSCLC) were initially diagnosed with distant metastasis. At present, there is no study to clarify the correlation between the primary location of the tumor and the metastasis pattern in advanced NSCLC. So we conducted this study to explored the relationship between the tumor primary location and metastasis pattern in stage IV NSCLC.

**Methods:**

A total of 19,295 eligible patients were identified from 2010 to 2012 in the SEER database. The main endpoint of our study was overall survival (OS). The survival curves were created by using the Kaplan–Meier method and compared by the usage of the Log Rank test. The clinical variable characteristics were compared by the chi-square test, and multivariate logistic regression analyses were used to evaluate the risk factors on metastasis patterns. All statistical *P* values were two-sided, and it was considered statistically significant when *P* ≤ 0.05.

**Results:**

We found that different proportions of metastatic sites could be found in different tumor primary locations. In addition, the prognosis of lung metastasis was relatively good in patients with tumor location in main bronchus (*P* < 0.001), upper lobe (*P* < 0.001), lower lobe (*P* < 0.001) , and middle lobe (*P* = 0.005). Besides, there was no significant OS difference for patients whose primary location was overlapping lesion (*P* = 0.226). The results also demonstrated that compared with patients with primary tumor located in the main bronchus, those in the upper lobe were more likely to have brain metastasis (*P* = 0.01) and lung metastasis (*P* = 0.024), those in the middle lobe were more prone to develop lung metastasis (*P* = 0.035) and those in the lower lobe were more apt to cause bone metastasis (*P* = 0.005) and lung metastasis (*P* = 0.001). In addition, there was no statistical difference in metastasis patterns among patients with overlapping lesions (*P* > 0.05).

**Conclusions:**

Different primary tumor locations might affect the metastasis pattern in patients with stage IV NSCLC.

## 1. Introduction

Lung and bronchus cancer is one of the most common types of malignant neoplasms as well as the leading cause of cancer mortality [1]. Nonsmall cell lung cancer (NSCLC) accounts for approximately 85% of all lung cancer cases [2], which representative histopathological types are adenocarcinoma (AD), squamous cell carcinoma (SCC), and large cell carcinoma [3]. The 5-year overall survival (OS) rates of early stage NSCLC patients was in the range of 40% to 70% following standard surgical treatment or stereotactic body radiation therapy. The expected survival rates at 5 years of patients with locally advanced disease using multidisciplinary treatment modalities were 15%–30%, while the 5-year OS rates for standard treatment based on platinum-doublet chemotherapy was less than 5% in advanced NSCLC [4, 5].

Most patients with NSCLC were initially diagnosed with distant metastasis [6, 7]. Bone metastasis was the most common metastasis type with approximately 40% of NSCLC cases, followed by metastasis of lungs, brain, and liver [8–11]. Although systemic therapy, surgical intervention, and radiotherapy had made some progress, patients with metastatic disease were still associated with poor prognosis [6, 12–14]. With the introduction of immunotherapy, the treatment of metastatic NSCLC has changed rapidly [15–18]. Pembrolizumab monotherapy was approved for first-line treatment of metastatic NSCLC, and the survival rate of metastatic NSCLC patients with negative gene mutations with high PD-L1 expression (tumor proportion score ≥50%) was significantly improved compared with double platinum chemotherapy [19]. A study showed that nivolumab provided long-term clinical benefits for patients with advanced NSCLC compared with docetaxel, with a 2-year survival rate of 23% in patients with squamous NSCLC and 29% in patients with nonsquamous NSCLC [20].

In esophageal cancer and colon cancer, studies [21, 22] had shown the connection between tumor primary location and metastasis site, and in completely resectable NSCLC, some studies [23, 24] had shown the association between primary tumor location and lymphatic vascular invasion. However, there is no study to clarify the correlation between the primary location of the tumor and the metastasis pattern in stage IV NSCLC. Therefore, we conducted this study to explore the relationship between the primary tumor location and metastasis pattern in stage IV NSCLC.

## 2. Materials and Methods

### 2.1. Patient Selection

The Surveillance Epidemiology and End Results (SEER) program, which is supported by the Surveillance Research Program in the National Cancer Institute's Division of Cancer Control and Population Sciences (DCCPS), provides information on cancer statistics in order to reduce the cancer burden among the US population [25]. We collected information of 19,295 appropriate patients from the SEER database and we screened the NSCLC patients with pathological diagnosis of adenocarcinoma and squamous cell carcinoma between 2010 and 2012 by the usage of SEER^*∗*^Stat 8.3.5 software. The inclusion criteria for this study were as follows: only one primary tumor, definite primary location, confirmed bone, brain, liver, or lung metastasis, active follow-up, and complete clinical information, such as age, race, sex, stage, and survival time.

### 2.2. Ethics Statement

This study was primarily based on the SEER database and did not require informed consent as personal identifying information was not included. Our study was approved by the ethics committee of the Shandong Cancer Hospital and was conducted in accordance with the Declaration of Helsinki. We accessed the data from the SEER database with reference number 12356-Nov2017.

### 2.3. Statistical Analysis

The main endpoint of our study was overall survival (OS). The survival curves were created by using the Kaplan–Meier method and compared by the usage of the Log Rank test. We compared the clinical variable characteristics through a Chi-square test and multivariate logistic regression analyses were used to evaluate the relationship between different prognostic factors and metastasis patterns. All statistical *P* values were two-sided and it was considered statistically significant when *P* ≤ 0.05. The Statistical Product and Service Solutions 22.0 (SPSS, IL, Chicago) software package was applied for all statistical analyses.

## 3. Results

### 3.1. Patients Characteristics

In this study, there were 11,360 (58.9%) patients who were ≥65 years old, and nearly three-quarters of patients were a white race. More than half of all patients were male gender. Among these patients, 13,745 (71.2%) were diagnosed with adenocarcinoma, and 5,550 (28.8%) were diagnosed with squamous cell carcinoma. The proportions of the *T*1, *T*2, *T*3, and *T*4 were 11.5%, 28.8%, 25.7%, and 34.0%, respectively. The highest proportion of *N* stage was *N*2, which accounted for 47.2%, while the lowest proportion was *N*1, which merely accounted for 8.2%. As for primary location, the main site was the upper lobe (59.1%), followed by the lower lobe (29.2%), and the rest were the main bronchus (5.5%), middle lobe (5.0%), and overlapping lesion (1.3%) successively. In addition, the number of patients with bone metastasis and lung metastasis was similar, and the least was liver metastasis. Detailed data are shown in Table 1.

### 3.2. Percentage of Metastatic Site Based on Different Primary Tumor Locations

**Figure 1:**
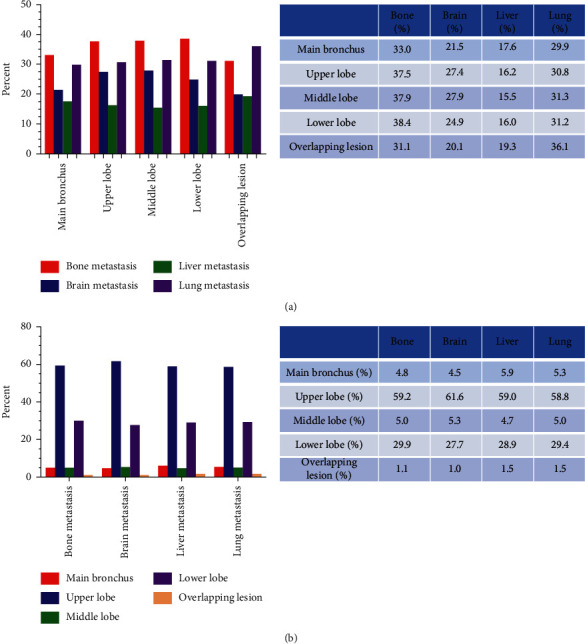
(a) The proportion of different metastasis patterns for patients with different primary locations. (b) The proportion of different primary locations for patients with different metastasis patterns.

As can be easily seen from the histogram (Figure 1(a)), if the primary location was the main bronchus, upper lobe, middle lobe, or lower lobe, the proportion of bone metastasis was highest and the proportion of liver metastasis was lowest while the primary location was overlapping lesion, with the highest percentage of lung metastasis, followed by bone metastasis, and the lowest percentage of liver metastasis. Turning the two factors in reverse, we could find details in Figure 1(b). Whether the site of metastasis was the bone, brain, lung, or liver, the primary location with the highest proportion was the upper lobe, followed by the lower lobe, and the lowest percentage was overlapping lesion. Besides, the proportion of the main bronchus and middle lobe was similar, only higher than that of the overlapping lesion.

### 3.3. Survival Difference of Metastasis Pattern Based on Different Primary Tumor Locations

**Figure 2:**
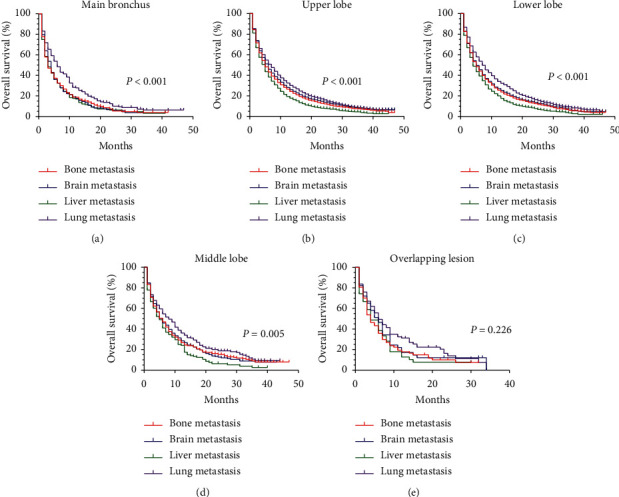
Survival difference for patients with specific primary location according to different metastasis patterns. Kaplan–Meier curves for patients with primary location in (a) the main bronchus based on different metastasis patterns; (b) the upper lobe based on different metastasis patterns; (c) the lower lobe based on different metastasis patterns; (d) the middle lobe based on different metastasis patterns; (e) overlapping lesions based on different metastasis patterns.

Survival curves were generated by the Kaplan–Meier analysis and compared by the usage of the Log Rank test. We found that patients with lung metastasis whose primary location was the main bronchus had a better OS (*P* < 0.001). The curves of patients with brain, liver, or bone metastasis, whose primary location was the main bronchus, almost overlapped (Figure 2(a)). For patients whose primary location was the upper lobe or lower lobe, the lung metastasis all had a relatively good prognosis while the liver metastasis had the worst OS (*P* < 0.001) (Figures 2(b) and 2(c)). We took the patients whose primary location was middle lobe (Figure 2(d)) into consideration and we could also find patients with lung metastasis had a comparatively good prognosis. However, the prognosis of patients with liver metastasis was the worst (*P*=0.005). The result of Figure 2(e) showed that there was no significant difference in OS for patients whose primary location was overlapping lesion (*P*=0.226).

### 3.4. Primary Tumor Location Could Be Used as Risk Factors on Metastasis Pattern

Multivariate logistic regression analysis was used to evaluate the relationship between tumor primary location and metastasis pattern. Detailed data are displayed in Table 2. The results showed that brain metastasis (OR 1.229; 95% CI 1.052–1.437; *P* = 0.01) and lung metastasis (OR 1.182; 95% CI 1.022–1.367; *P* = 0.024) were more likely to occur when the primary site was in the upper lobe than in the main bronchus, but there was no statistical difference between bone metastasis (*P* = 0.057) and liver metastasis (*P* = 0.463). Patients with primary site in the middle lobe were more prone to develop lung metastasis (OR 1.242; 95% CI 1.015–1.519; *P* = 0.035) than those in the main bronchus, while there was no difference in bone metastasis (*P* = 0.139), brain metastasis (*P* = 0.062), and liver metastasis (*P* = 0.384). In addition, the results also indicated that bone metastasis (OR 1.222; 95% CI 1.062–1.408; *P* = 0.005) and lung metastasis (OR 1.299; 95% CI 1.116–1.512; *P* = 0.001) more tended to the occurrence of tumors with the primary site in the lower lobe than those located in the main bronchus. However, there was no such trend in brain metastasis (*P* = 0.149) and liver metastasis (*P* = 0.550). At the same time, we found that there was no difference in bone metastasis (*P* = 0.342), brain metastasis (*P* = 0.252), liver metastasis (*P* = 0.495), and lung metastasis (*P* = 0.402) in patients whose primary location was overlapping lesion.

## 4. Discussion

In this study, we explored the prognostic significance of primary tumor location and metastasis pattern in patients with stage IV NSCLC. Meanwhile, we elucidated the prognostic relevance between both. The results demonstrated that the prognosis of lung metastasis was relatively good in patients with tumor location in the main bronchus, upper lobe, middle lobe, and lower lobe, while the prognosis of liver metastasis was the worst. Compared with patients with primary tumor located in the main bronchus, those in the upper lobe were more likely to have brain and lung metastasis, those in the middle lobe were more prone to develop lung metastasis, and those in the lower lobe were more apt to develop bone and lung metastasis. In addition, there was no statistical difference in metastasis patterns among patients with overlapping lesions.

It had been proved that the metastatic site of NSCLC was related to the primary site [23, 24]. Grbić et al. [23] found that the rate of hilar lymph node metastasis in central lung cancer was significantly higher than that in peripheral lung cancer, and the incidence of lymphatic vascular invasion was the most common in upper lobe tumors. Kotoulas et al. [24] conducted a retrospective study of 557 patients and found that the metastatic site of the tumors in the upper lobe, middle lobe, and lower lobe was different in patients who could be resectable. To some extent, these results supported us in carrying out this study.

A large cohort analysis indicated primary tumor location (main bronchus or nonmain bronchus) played an important role in predicting the metastasis site of lung adenocarcinoma (ADC), which showed that main bronchus location was a predictor of lung ADC metastasis and prognosis [26]. Research in Taiwan also proved that tumors located in the main bronchus were considered to be an independent prognostic factor [27]. For patients undergoing resection, the prognosis of patients with main bronchus tumors was worse than that of patients with nonmain bronchus tumors, which might be explained by special anatomical structures, such as sleeve lobectomy [26, 28]. Besides, Wang et al. [27] found patients with tumors located in multiple lobes presented as the worst prognosis. It could be that the survival time of patients with overlapping lesions was too short to distinguish the metastatic mode of patients whose primary location of the tumor was the overlapping lesion.

A study had constructed a nomogram that can predict the risk factors of liver and lung metastasis in patients with colon cancer, in which tumor location was an independent risk factor for metastasis [21]. In esophageal cancer, tumor location was also an independent risk factor for metastasis. The results showed that lung metastasis was more likely to occur in the upper segment of esophageal cancer than in the lower segment (*P*=0.033), while liver metastasis was more apt to occur in the lower segment than in the upper segment (*P*=0.014) [22]. The anatomical hypothesis might partly explain the difference in metastatic sites caused by different tumor locations. Our research coincided with the purpose of these studies to predict metastasis risk and prognosis through the primary site in order to avoid unnecessary treatment delays and make full use of medical resources.

Several studies reported liver metastasis was associated with impaired survival [12, 29, 30]. A retrospective study [29] found liver metastasis was the worst prognostic factor in OS and cancer-specific survival. One research [30] that involved 148 patients showed liver metastasis predicted poorer progression-free survival (PFS) and OS in stage IV lung adenocarcinoma patients who received gefitinib as first-line therapy. Topalian et al. [31] demonstrated that the presence of liver metastasis was independently associated with a reduced 5-year survival rate in patients treated with nivolumab. Tumeh et al. [32] found that liver metastasis was associated with reduced marginal CD8^+^*T* cell infiltration, which might be a potential mechanism for reduced response to immune checkpoint inhibitors (ICIs) monotherapy. In contrast, a meta-analysis [33] showed that there was no significant correlation between liver metastasis in patients with advanced lung cancer and the efficacy of immunotherapy combined with chemotherapy as first-line therapy; in other words, patients with and without liver metastasis in advanced lung cancer could get similar benefits from this treatment. Unfortunately, the specific mechanism of this phenomenon is still unclear. The most common pathogenesis of hepatocellular carcinoma was chronic viral infections. Thus, differences in tumor biology might be a reason for poor survival. In addition, mutation heterogeneity of tumor could also be a reasonable explanation for this result, as the primary location and the metastatic site had different mutation profiles [34]. This might explain why there was no difference in the occurrence of liver metastasis at different primary sites.

The advantage of our study is its large population size, which leads to enough power to detect small differences in the results. However, this study had certain limitations. Firstly, as retrospective research of metastatic NSCLC cases, it had its intrinsic shortcomings. Secondly, the information about the therapeutic regimen and progression of metastatic disease was absent. Thirdly, some other variables that might affect survival and prognosis could not be extracted from the SEER database, such as smoking history, performance status, tumor differentiation, and laboratory parameters. In addition, gene mutations were not covered in this database, which might be related to different metastasis patterns and prognosis. Furthermore, we only collected information about lung, liver, brain, and bone metastases but did not count other metastatic patterns, such as adrenal gland, which may lead to the underestimation of other metastatic patterns. Therefore, large-scale prospective studies are needed to further clarify these results.

In summary, different primary tumor sites might affect the metastasis pattern of patients with stage IV NSCLC and the prognosis of lung metastasis was relatively good in patients with tumor location in the main bronchus, upper lobe, middle lobe, and lower lobe. The primary site of the tumor was used to predict the metastatic risk and prognosis of patients so as to avoid unnecessary delays in treatment and make full use of medical resources. In addition, prospective studies are needed to fully verify the role of primary tumor location in predicting metastatic patterns.

## Figures and Tables

**Table 1:** Characteristics of patients from SEER database according to different variables.

Variables	Number	%
Age		
<65	7935	41.1
≥65	11,360	58.9

Race		
White	14,931	77.4
Black	2685	13.9
Others	1679	8.7

Sex		
Female	8571	44.4
Male	10,724	55.6

Histology		
Adenocarcinoma	13,745	71.2
Squamous cell carcinoma	5550	28.8

*T* stage		
*T*1	2211	11.5
*T*2	5558	28.8
*T*3	4964	25.7
*T*4	6562	34.0

*N* stage		
*N*0	4712	24.4
*N*1	1591	8.2
*N*2	9110	47.2
*N*3	3882	20.1

Primary site		
Main bronchus	1054	5.5
Upper lobe	11,401	59.1
Middle lobe	956	5.0
Lower lobe	5640	29.2
Overlapping lesion	244	1.3

Bone metastasis		
Yes	7230	37.5
No	12,065	62.5

Brain metastasis		
Yes	5074	26.3
No	14,221	73.7

Liver metastasis		
Yes	3129	16.2
No	16,166	83.8

Lung metastasis		
Yes	5976	31.0
No	13,319	69.0

**Table 2:** Multivariate logistic regression analyses to evaluate the risk factors for different metastasis patterns.

Variables	Bone metastasis		Brain metastasis		Liver metastasis		Lung metastasis	
	OR (95% CI)	*P*	OR (95% CI)	*P*	OR (95% CI)	*P*	OR (95% CI)	*P*
Age		<0.001		<0.001		0.060		<0.001
<65	Reference							
≥65	0.860 (0.810–0.914)	<0.001	0.581 (0.544–0.621)	<0.001	0.927 (0.857–1.003)	0.060	1.222 (1.143–1.307)	<0.001

Race		<0.001		0.088		0.239		<0.001
White	Reference							
Black	0.834 (0.764–0.911)	<0.001	0.911 (0.827–1.003)	0.057	0.913 (0.814–1.023)	0.116	1.011 (0.920–1.112)	0.817
Others	0.991 (0.893–1.101)	0.871	1.051 (0.938–1.178)	0.390	0.944 (0.821–1.085)	0.415	1.277 (1.141–1.429)	<0.001

Sex		<0.001		<0.001		0.064		0.028
Female	Reference							
Male	1.188 (1.119–1.261)	<0.001	0.886 (0.829–0.946)	<0.001	1.077 (0.996–1.164)	0.064	0.929 (0.870–0.992)	0.028

Histology		<0.001		<0.001		0.794		<0.001
Adenocarcinoma	Reference							
Squamous cell carcinoma	0.663 (0.619–0.709)	<0.001	0.501 (0.461–0.543)	<0.001	1.012 (0.928–1.103)	0.794	0.849 (0.789–0.914)	<0.001

*T* stage		<0.001		<0.001		0.206		<0.001
*T*1	Reference							
*T*2	0.847 (0.765–0.937)	0.001	0.980 (0.878–1.094)	0.721	1.059 (0.921–1.219)	0.421	1.408 (1.210–1.637)	<0.001
*T*3	0.815 (0.734–0.904)	<0.001	0.843 (0.752–0.944)	0.003	1.131 (0.982–1.303)	0.088	4.400 (3.808–5.086)	<0.001
*T*4	0.829 (0.750–0.917)	<0.001	0.794 (0.711–0.886)	<0.001	1.131 (0.986–1.297)	0.079	6.967 (6.048–8.024)	<0.001

*N* stage		<0.001		<0.001		<0.001		<0.001
*N*0	Reference							
*N*1	1.279 (1.135–1.440)	<0.001	1.194 (1.047–1.361)	0.008	1.304 (1.110–1.532)	0.001	0.915 (0.796–1.050)	0.205
*N*2	1.314 (1.219–1.417)	<0.001	1.172 (1.078–1.273)	<0.001	1.510 (1.362–1.673)	<0.001	1.120 (1.030–1.218)	0.008
*N*3	1.308 (1.195–1.432)	<0.001	1.031 (0.932–1.141)	0.552	1.473 (1.304–1.664)	<0.001	1.654 (1.500–1.823)	<0.001

Primary site		0.009		0.003		0.723		0.008
Main bronchus	Reference							
Upper lobe	1.141 (0.996–1.307)	0.057	1.229 (1.052–1.437)	0.010	0.939 (0.794–1.111)	0.463	1.182 (1.022–1.367)	0.024
Middle lobe	1.150 (0.956–1.384)	0.139	1.219 (0.990–1.501)	0.062	0.900 (0.709–1.141)	0.384	1.242 (1.015–1.519)	0.035
Lower lobe	1.222 (1.062–1.408)	0.005	1.127 (0.958–1.326)	0.149	0.948 (0.795–1.130)	0.550	1.299 (1.116–1.512)	0.001
Overlapping lesion	0.864 (0.639–1.169)	0.342	0.814 (0.573–1.157)	0.252	1.132 (0.792–1.618)	0.495	1.139 (0.840–1.544)	0.402

## Data Availability

The raw data used to support the results of this study can be obtained from the corresponding author upon reasonable request.
